# Tungsten Alloy and Cancer in Rats: Link to Childhood Leukemia?

**DOI:** 10.1289/ehp.113-a801

**Published:** 2005-12

**Authors:** John D. Schell

**Affiliations:** Blasland, Bouck & Lee, Inc., Houston, Texas, E-mail: js1@bbl-inc.com

We read with interest the article by Kalinich et al. (2005) on the generation of rhabdomyosarcomas from “embedded weapons-grade tungsten alloy.” Although the study design and the reported findings are of great interest, we are concerned about certain statements made in both the “Introduction” and the “Discussion” of the article. In these sections the authors make reference to the allegation that “several cancer clusters in the United States are associated with elevated levels of tungsten in the environment” (Kalinich et al. 2005) Although they accurately point out that “no definitive link … has been established,” they suggest that the cancer clusters are part of “a growing list of health concerns related to tungsten exposure.” However, the conditions at Fallon, Nevada, and the investigations into a purported link between naturally occurring tungsten and childhood leukemia are very different from the experimental conditions that exist in the implantation study by Kalinich et al. (2005).

The Centers for Disease Control and Prevention (CDC) conducted a thorough investigation into the Fallon cancer cluster; in fact, it was the largest cancer cluster investigation ever undertaken in the United States. The scientists from the CDC and state health departments concluded that exposure to tungsten was not associated with the incidence of childhood leukemia in Fallon (CDC 2003). The genesis of the leukemia cases is still an area of interest and speculation as shown by a recent letter in *EHP* (Daughton 2005). Because Kalinich et al. (2005) inferred that tungsten somehow played a role in the Fallon leukemias while presenting data suggesting that implanted tungsten alloy caused metastatic tumor formation, readers may confuse the issues and assume that somehow the two effects (rhabdomyosarcoma and childhood leukemia) are related.

We are not questioning the quality of the work presented by Kalinich et al. (2005) or their finding that implanted pellets of a specific combination of tungsten/nickel/cobalt alloy caused an apparent increase in rhabdomyosarcoma with subsequent metastasis to the lung. Rather, we recommend that the authors remain focused on this finding. Suggesting that these results can be linked to, or somehow shed light on, childhood leukemia and exposure to environmental tungsten is both inappropriate and misleading.
